# Vogt–Koyanagi–Harada disease-like uveitis following nivolumab administration treated with steroid pulse therapy: a case report

**DOI:** 10.1186/s12886-020-01519-5

**Published:** 2020-06-24

**Authors:** Ryo Kikuchi, Tatsukata Kawagoe, Kazuki Hotta

**Affiliations:** 1 Department of Ophthalmology, Kameda Medical Center, Kamogawa, Japan; 2 Department of Ophthalmology and Visual Science, Yokohama City University School of Medicine, Yokohama, Japan

**Keywords:** Immune checkpoint inhibitors, PD-1 inhibitor, Nivolumab, Immune related adverse events, Vogt–Koyanagi–Harada disease, HLA-DR4, Examination of cerebrospinal fluid, Steroid pulse therapy

## Abstract

**Background:**

Immune checkpoint inhibitors can cause various adverse effects. Recently it has been shown that Vogt–Koyanagi–Harada (VKH) disease-like uveitis can occur in patients treated with nivolumab.

**Case presentation:**

A 69-year-old man developed bilateral panuveitis after nivolumab treatment for recurrent hypopharyngeal cancer. Slit lamp examination revealed bilateral granulomatous keratic precipitates, anterior chamber cells and partial synechiae. Fundus examination revealed bilateral optic disc edema and diffuse serous retinal detachment. His human leukocyte antigen (HLA) typing showed HLA-DRB1*04:05 allele. A lumbar puncture did not demonstrate pleocytosis. Bilateral sub-tenon injections of triamcinolone acetonide were initiated. As his panuveitis did not regress completely, steroid pulse therapy was administered. That therapy led to the resolution of his serous retinal detachment and to rapid improvement in his vision. Following this, we treated him with 50 mg/day of prednisolone for 1 week and then reduced it by 5 mg every week. No bilateral uveitis relapse had occurred by his 3-month follow-up; however, he subsequently died because of his cancer.

**Conclusion:**

To our knowledge, this is the first report of a patient with NVKH who underwent a lumbar puncture. Unlike VKH, our case did not show meningismus or pleocytosis. NVKH may, therefore, have a different etiology from VKH.

In cases of NVKH with posterior uveitis, steroid pulse therapy may be considered as a treatment option, as it is in VKH.

## Background

Programmed Death-1 (PD-1) is a cell-surface receptor whose ligands, PD-L1 and PD-L2, inhibit T cell activation. PD-L1 is expressed in many tumor and immune cells. PD-L1 in tumor cells can evade the host immune system. Nivolumab (Opdivo®), a fully human IgG4 monoclonal antibody, binds to PD-1 and blocks the interaction with PD-L1. The cancer cells are thus exposed to the immune system by activated T cells. Compared with standard therapy, nivolumab was associated with significant improvements in overall survival among previously untreated patients who had metastatic melanoma without a BRAF mutation [1]. Among patients with platinum-refractory, recurrent squamous-cell carcinoma of the head and neck, treatment with nivolumab resulted in longer overall survival than treatment with standard single-agent therapy [2]. Immune checkpoint inhibitors, including nivolumab, stimulate the immune system and cause various autoimmune-like side effects, which are known as immune-related adverse events (irAEs). In ophthalmology, uveitis and dry eye are among the known irAEs [3]. Recently, the development of VKH-like uveitis after nivolumab administration was identified for the first time, but this has not been investigated in detail. We report a case of a patient with bilateral NVKH who underwent cerebrospinal fluid (CSF) examination and treatment with steroid pulse therapy.

## Case presentation

A 63-year-old man was diagnosed with hypopharyngeal cancer (Stage IVb) affecting the lymph nodes in July 2012. Complete remission was achieved through a combination of chemotherapy and radiotherapy. However, the hypopharyngeal cancer recurred in August, 2017 and he underwent resection of the larynx, pharynx, esophagus, thyroid and cervical lymph nodes in September 2017. Lymph node metastasis appeared in December 2017, and nivolumab was administered. Prior to presentation to us, he had received two cycles of nivolumab treatment at a dose of 160 mg (3 mg/kg body weight) on February 16 and March 2, 2018. A third treatment was discontinued owing to his deteriorating health.

**Fig. 1:**
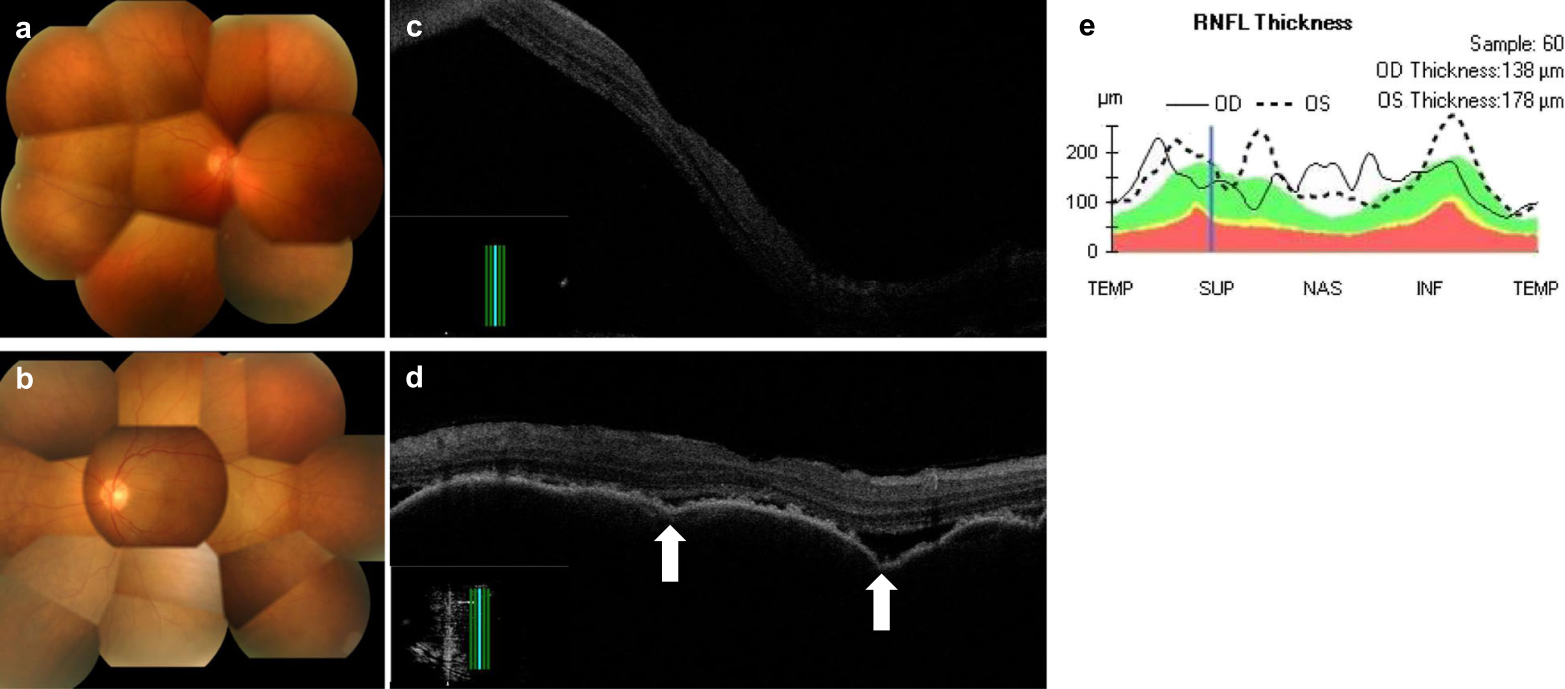
Color fundus photographs and optical coherence tomography (OCT) images of the right (**a** and **c**) and left (**b** and **d**) eyes at presentation. OCT image of retinal nerve fiber layer thicknesses (**e**) The diffuse serous retinal detachment, retinal pigment epithelium folds, and optic disc swelling are shown. Arrows indicate peaks of the retinal pigment epithelium folds

**Fig. 2:**
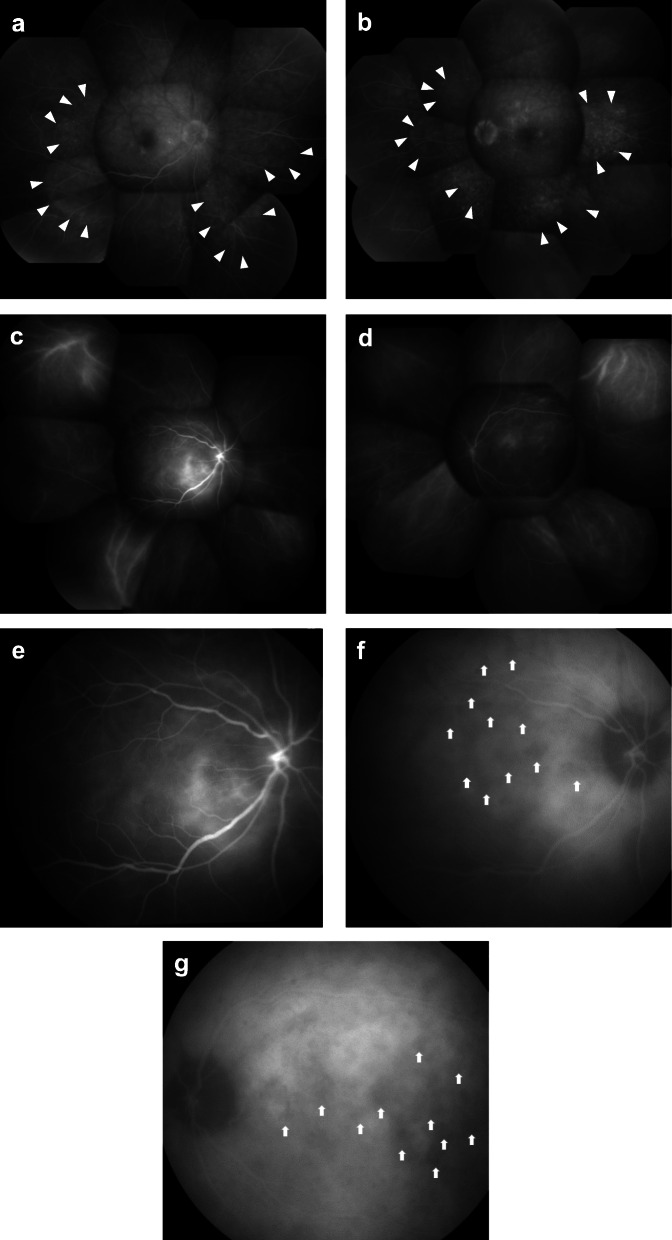
Fluorescein angiography and indocyanine-green angiography before systemic steroid treatment. Fluorescein angiography demonstrated multiple small leaks from the fundus in the intermediate phase (**a** and **b**). Arrowheads indicate leakages Indocyanine-green angiography also demonstrated leakage (**c** and **d**). Delayed choroidal perfusion is apparent in the early angiographic phase (31 s) (**e**). Hypofluorescent dark dots are visible in the intermediate phase (12 min) (**f** and **g**). Arrows point to the hypofluorescent dark dots

He was referred to our clinic with a complaint of blurry vision starting 6 days prior to presentation on April 21, 2018. He did not have tinnitus, meningismus or vitiligo. His past ocular history was unremarkable. At presentation, his best-corrected visual acuity was 10/200 in the right eye and 20/50 in the left eye. Slit lamp examination revealed bilateral anterior uveitis. There were granulomatous mutton-fat keratic precipitates and anterior chamber cells. Both irises exhibited posterior synechiae. Fundus examination showed papilledema and serous retinal detachment in both eyes (Fig. 1, a, b). Vitreous haze was not observed in either eye. Optical coherence tomography showed subretinal fluid, a wavy retinal pigment epithelium line and optic disc swelling (Fig. 1, c–e). Fluorescein angiography demonstrated leakages of fundi and optic disc (Fig. 2, a, b, and e). Indocyanine green angiography demonstrated leakage (Fig. 2, c and d), the incompetency of the choroidal circulation (Fig. 2, f) and hypofluorescent dark dots in the intermediate phase (Fig. 2, g, h). His genotyping showed HLA-DRB1*04:05. However, examination of CSF did not show pleocytosis. He was diagnosed with probable VKH, based on the revised diagnostic criteria for VKH [4]. We suspected nivolumab as the cause of the uveitis.

**Fig. 3:**
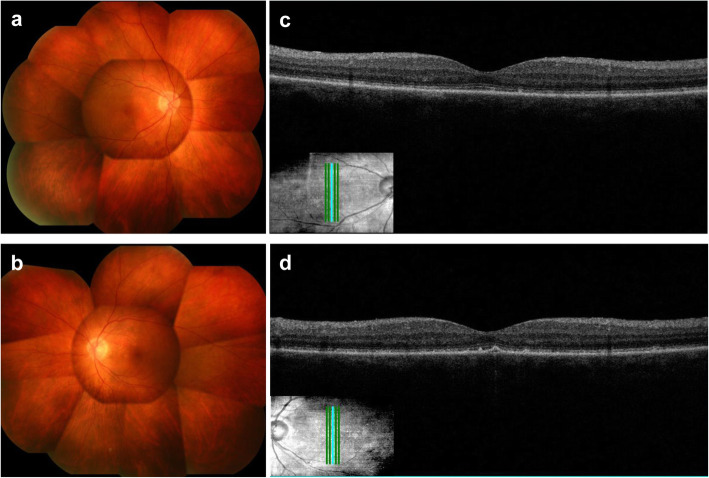
Color fundus photographs and optical coherence tomography images after steroid pulse therapy. The fundus findings had improved in both eyes. The fundus photographs show the sunset glow appearance

In consideration of the patient’s systemic condition, bilateral sub-tenon injection of triamcinolone acetonide (STTA) was initiated as the primary therapy on April 27. Because this did not achieve remission, we started steroid pulse therapy on May 2. We intravenously administered 1000 mg/day of methylprednisolone sodium succinate for 3 days, which led to a rapid improvement in the patient’s vision and resolution of the anterior uveitis and serous retinal detachment (Fig. 3, a–d). After this therapy finished, we treated him with 50 mg/day of prednisolone for 1 week, and then reduced it by 5 mg every week. The patient’s uveitis remained in complete remission at his last visit on June 28. The uveal inflammation had subsided, and depigmentation and a sunset glow appearance were observed in both fundi. The patient died from cancer on August 4.

## Discussion and conclusions

Immune checkpoint inhibitors have revolutionized cancer treatment, but irAE side effects are known to occur. IrAEs include uveitis, orbital inflammation, rash, vitiligo, dry mouth, enterocolitis, hypothyroidism, hypophysitis, hepatitis, pneumonitis, pancreatitis, autoimmune diabetes, polyarthritis, adrenal insufficiency, myasthenia gravis and systemic lupus erythematosus [5].

To the best of our knowledge, there have only been five published case reports from six NVKH patients until now [6–10] (Table 1).

**Table 1:** Previous reports of nivolumab-related Vogt–Koyanagi–Harada (NVKH)-like uveitis

Authors	Age Sex	Neurological and auditory symptoms	Pleocytosis	HLA classII examnation	Diagnosis	Treatment
Our case	69 M	N	N	DRB1*04:05 DRB1*09:01	bilateral VKHD like panuveitis	
Arai et al. [6]	55 M	N	–	DRB1*04:10 DRB1*04:06	bilateral anterior uveitis	
Matsuo et al. [7]	60 F	Headache	–	–	bilateral VKHD like posterior uveitis	
Fujimura et al. [8]	73 M	Hearing loss	–	DRB1*04:05	VKHD like uveitis	
	35 F	Hearing loss Headache	–	DRB1*04:05	VKHD like uveitis	
Wang et al. [9]	64 F	–	–	–	bilateral panuveitis	▪ Topical and oral steroids ▪ Intravenous and periorbital injection of methylprednisolone ▪ Intravitreal dexamethasone implant
Obata et al. [10]	63 F	–	–	–	bilateral VKHD like panuveitis	▪ Topical corticosteroid

*F* Female, *M* Male, *N* Negative, −: not described

Arai et al. reported the case of a 55-year-old man with acute anterior uveitis [6]. Slit lamp examination showed inflammatory reaction with fibrin formation and posterior synechiae in both eyes. Two months later, poliosis and alopecia areata emerged. Neurological and auditory symptoms were not observed. The patient had HLA-DRB1*04:10 and HLA-DRB1*04:06 alleles. Because the uveitis was confined to the anterior of the eye, it was controlled by steroid instillation.

Matsuo et al. reported the case of a 60-year-old woman with posterior VKH-like uveitis that occurred following the administration of nivolumab and vemurafenib (BRAF inhibitor) [7]. She also had headaches and general arthralgia and systemic skin rashes. HLA analysis was not performed. Optical coherence tomography showed a wavy retinal pigment epithelium line with multifocal choroidal thickening in both eyes, together with subretinal fluid only in the right eye. In this case, nivolumab and vemurafenib were discontinued temporarily, and 0.1% betamethasone eye drops and oral prednisolone (tapered from 30 mg) were used. The elevated bumps disappeared from both retinae. Vemurafenib and nivolumab were re-administered, and intraocular inflammation recurred 2 months later. She resumed 0.1% betamethasone eye drops, leading to the resolution of intraocular inflammation.

Fujimura et al. reported two cases of VKH-like posterior uveitis [8]. A 73-year-old man demonstrated bilateral serous retinal detachment and sensorineural hearing loss in the right ear about 4 months after nivolumab administration. He was also shown to carry the HLA-DRB1*04:05 allele. He was administered intravenous steroid pulse therapy and then treated with oral prednisolone. One week later his visual acuity had improved. A 35-year-old woman demonstrated bilateral serous retinal detachment, headache, and hearing loss. She was shown to carry the HLA-DRB1*04:05 allele. She also received steroid pulse therapy, and 1 week later her visual acuity had improved.

Wang et al. reported on a 64-year-old female patient with panuveitis and bilateral serous retinal detachment following treatment with nivolumab [9]. Anterior chamber cells and keratic precipitates were present in both eyes. HLA analysis was not performed. Treatment consisted of intravenous methylprednisolone, oral prednisone, topical steroid eye drops, periorbital injection of steroids and, finally, intravitreal injection of a steroid implant. The ocular inflammation was well-controlled. No neurological or auditory symptoms were mentioned in the report.

Obata et al. reported the case of a 63-year-old female patient with panuveitis and serous retinal detachment in both eyes [10]. The patient also complained of headaches. HLA analysis was not performed. Because the patient’s general condition was poor, nivolumab was discontinued and a topical corticosteroid was initiated, which controlled her uveitis.

VKH is reported to be closely associated with HLA-DR4, particularly HLA-DRB1*04:05 [11]. Some reports, including our case, have demonstrated a relationship between NVKH and HLA-DRB1*04:05. HLA-DRB1*04:05 may be important as one of the causes of NVKH.

Lumbar punctures were not performed in any of the cases reported above, so definite diagnoses of complete VKH were not made. However, neurological and auditory symptoms are often observed in, and associated with, VKH. Keino et al. investigated a total of 102 VKH patients and found that the frequency of CSF pleocytosis was 82.7%, of headache was 42.1% and of tinnitus was 31.3% [12]. In contrast, in seven NVKH patients (our case and the six earlier cases), there were only three (43%) with headache, two (29%) with hearing loss, and no cases of tinnitus. A CSF examination is recommended for the diagnosis of VKH owing to the test’s high sensitivity. Our case is the first report that used a CSF examination in NVKH. Unexpectedly, our patient did not show pleocytosis and was diagnosed as probable VKH according to the revised diagnostic criteria for VKH [4]. It is rare that neurological and/or auditory symptoms, or pleocytosis are not detected in VKH. Therefore, it is possible that NVKH has a different etiology from VKH.

The mechanism of VKH remains unknown, but it is assumed to be an autoimmune disease involving melanocytes [13]. Nivolumab blocks PD-L1, and T cells specific for melanocytes may be activated. The melanocyte-specific antigen may be presented by HLA-DR4 positive antigen-presenting cells to activated T cells. T cells activated by nivolumab may not have high affinity with meninges less than T cells in VKH.

Most irAEs are reversible with steroids, provided that the treatment is initiated early and at a sufficient dosage level. However, no effective therapy for NVKH has been established to-date. Despite his severe panuveitis, we decided to initiate STTA owing to our patient’s general condition. We did not achieve complete remission with STTA, and subsequently administered steroid pulse therapy. Steroid pulse therapy led to complete remission throughout the prolonged observation period. Fujimura et al. also showed that steroid pulse therapy improved severe serous retinal detachment after nivolumab treatment [8]. Therefore, steroid pulse therapy might be the optimum first-line therapy for both VKH and NVKH, especially for severe posterior uveitis cases.

Knowledge about VKH-like uveitis associated with nivolumab is increasing. HLA-DRB1*04:05 may be one of the causes of both NVKH and VKH. Unlike VKH, our patient did not show meningismus or pleocytosis. Further cases are necessary to illuminate the relationship between CSF and NVKH.

Recently, it has been learned that other immune checkpoint inhibitors such as anti-PD-1 antibody pembrolizumab and anti-PD-L1 antibody atezolizumab are also capable of causing VKH-like uveitis [14, 15]. It is expected that further reports about these drugs will elucidate the mechanism of immune checkpoint inhibitor-induced diseases.

In posterior uveitis cases of NVKH, steroid pulse therapy might be a first-line therapy. Further reports are needed to improve methods for determining the diagnosis and to determine the optimal treatment regimen for this disease.

## Data Availability

Not applicable.

## Abbreviations

VKH: Vogt–Koyanagi–Harada disease
NVKH: Nivolumab-related Vogt–Koyanagi–Harada-like disease
HLA: Human leukocyte antigen
